# Supplementary material for: Systematic Review and Meta-Analysis of Transcendental Meditation for Post-Traumatic Stress Disorder
Source: Medicina (Kaunas). 2025 Apr 3;61(4):659. doi: 10.3390/medicina61040659 (PMC12028398; doi:10.3390/medicina61040659)
Supplement: Supplementary file 1 [file medicina-61-00659-s001.zip › Supplementary Materials File S1, Flow Diagram and Literature Search-medicina-3381954.pdf]

Flow Diagram and Description of Literature Search

Screening. Two authors (DOJ, JT) working independently conducted the data searches, with updated searches made in 2020, 2021, 2022, 2023, and 2024. Two authors (DOJ, BR) independently coded studies for implementation statistics. Two authors (DOJ and VB) independently coded studies for research quality on the modified Clear Score scale. Data for calculating effect sizes and meta-regression was coded by DOJ and independently proofed by Rhoda Orme-Johnson, a professional proofreader. Any discrepancies between coders were discussed and resolved with reference to the original text in the papers.

Literature Search Flow Diagram.

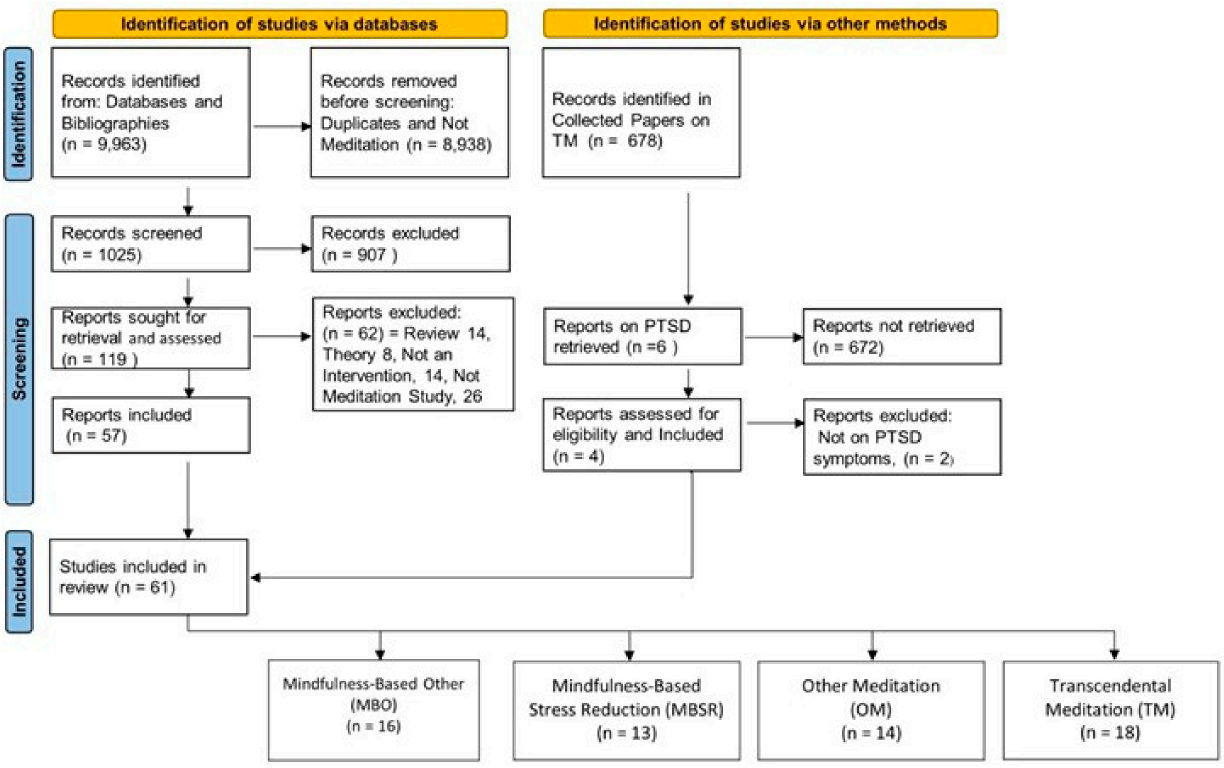

Flow diagram of literature search.

Data bases (MEDLINE, PubMed, PsycINFO, Web of Science, Library of Congress, and Google Scholar) were searched for keywords “Mindfulness-Based Stress Reduction”, “MBSR”, “Transcendental Meditation”, “TM”, “mindfulness”, and “meditation” from January 1970 to June 2024. These searches identified 9,963 records on meditation. Of these, 8,938 were excluded because they were not on PTSD or trauma or otherwise did not meet our study inclusion criteria. Titles and abstracts of 1025 records were obtained for screening, of which 907 were excluded, leaving 119 papers sought for retrieval and assessment for eligibility. The 119 records assessed for eligibility resulted in 62 records being excluded, because 14 were reviews, 8 were theoretical papers, and 14 records were not intervention studies on meditation. For example, they were feasibility studies on the use of meditation to treat PTSD or were cross sectional studies such as on the rate of PTSD in different populations or were correlational studies between the level of PTSD in people with different psychological or psychosocial characteristics, such as differences in qualities of mindfulness. Such studies, although of interest, did not address the issue of the efficacy of meditation as a treatment for PTSD and thus were excluded. The 26 others of the 118 were excluded because they were studies of the effects of other types of interventions in treating PTSD rather than meditation, such as trauma focus techniques, cognitive processing, hypnosis, hatha yoga, pharmaceuticals, or games. This left 57 studies from the searches of data bases, which with the addition of 4 TM studies identified via other sources, i.e., the Collected Papers on TM, resulted in a total of 61 studies included in our review.

Collected Papers on TM, which is on the righthand column in the flow diagram, refers to Volumes 1-8 of Scientific Research on the Transcendental Meditation and TM-Sidhi programs 23-28. These volumes, which span 1970 to 2013, contain 678 records. Of these, six records were on “PTSD” or “posttraumatic”, four met the inclusion criteria of a longitudinal intervention study

on PTSD, which added to the 57 studies identified via databases, resulted in 61 studies included in our meta-analysis.

These 61 studies were then logically divided into four categories of meditation, including the two categories that are uniformly defined, Transcendental Meditation (TM) and Mindfulness-Based Stress Reduction (MBSR). The first of the other two categories was Mindfulness-Based Other (MBO), which are mindfulness-based techniques but not MBSR. MBO techniques were often derived from MBSR but did not follow the MBSR protocol. MBO techniques often experimented with different lengths of training, mode of presentation (such as via telecommunications), or with additional techniques, such as Hatha Yoga. The fourth category of meditation was Other Meditations (OM), which were neither TM nor MBSR nor MBO. Examples of OM include compassion meditation, Kriya yoga, Adopted Mantra Meditation, and Mantrum Repetition meditation.

Data were then extracted from these 61 papers on study characteristics, such as experimental design, type of treatment, number of subjects, type of trauma, location of treatment, etc. Implementation statistics were also coded, such as the number of people offered meditation, number and percent who learned, number and percent who were post tested, etc. In addition, data from the papers needed for calculating effect sizes were entered into the meta-analysis and multivariate regression analysis program35. The above figure displays the results of our literature search, which located 61 studies that used meditation to treat PTSD, 16 studies on MBO, 13 on MBSR, 14 on OM, and 18 on TM.
